# Distinct responses of airborne abundant and rare microbial communities to atmospheric changes associated with Chinese New Year

**DOI:** 10.1002/imt2.140

**Published:** 2023-10-11

**Authors:** Hu Li, You‐Wei Hong, Meng‐Ke Gao, Xin‐Li An, Xiao‐Ru Yang, Yong‐Guan Zhu, Jin‐Sheng Chen, Jian‐Qiang Su

**Affiliations:** ^1^ Key Laboratory of Urban Environment and Health, Fujian Key Laboratory of Watershed Ecology, Institute of Urban Environment Chinese Academy of Sciences Xiamen China; ^2^ University of Chinese Academy of Sciences Beijing China; ^3^ CAS Center for Excellence in Regional Atmospheric Environment, Institute of Urban Environment Chinese Academy of Sciences Xiamen China; ^4^ College of Resource and Environmental Science Fujian Agriculture and Forestry University Fuzhou China; ^5^ State Key Lab of Urban and Regional Ecology, Research Center for Eco‐environmental Sciences Chinese Academy of Sciences Beijing China

**Keywords:** atmospheric environment, bacteria, community assembly, deterministic process, fungi, stochastic process

## Abstract

Airborne microorganisms, including pathogens, would change with surrounding environments and become issues of global concern due to their threats to human health. Microbial communities typically contain a few abundant but many rare species. However, how the airborne abundant and rare microbial communities respond to environmental changes is still unclear, especially at hour scale. Here, we used a sequencing approach based on bacterial 16S rRNA genes and fungal ITS2 regions to investigate the high time‐resolved dynamics of airborne bacteria and fungi and to explore the responses of abundant and rare microbes to the atmospheric changes. Our results showed that air pollutants and microbial communities were significantly affected by human activities related to the Chinese New Year (CNY). Before CNY, significant hour‐scale changes in both abundant and rare subcommunities were observed, while only abundant bacterial subcommunity changed with hour time series during CNY. Air pollutants and meteorological parameters explained 61.5%−74.2% variations of abundant community but only 13.3%−21.6% variations of rare communities. These results suggested that abundant species were more sensitive to environmental changes than rare taxa. Stochastic processes predominated in the assembly of abundant communities, but deterministic processes determined the assembly of rare communities. Potential bacterial pathogens during CNY were the highest, suggesting an increased health risk of airborne microbes during CNY. Overall, our findings highlighted the “holiday effect” of CNY on airborne microbes and expanded the current understanding of the ecological mechanisms and health risks of microbes in a changing atmosphere.

## Highlights

- Hour‐scale dynamics of airborne bacteria and fungi were determined.
- Human activities during Chinese New Year changed the distribution of air pollutants.
- Environmental changes during Chinese New Year (CNY) affected communities of airborne bacteria and fungi, distinctly.
- Abundant species were more sensitive than rare taxa in response to changes during CNY.
- Human activities during CNY increase the threats of airborne microbes to human beings.

## Graphical abstract

The hour‐scale dynamics of bacteria and fungi in the atmosphere were determined. Human activities during Chinese New Year changed the distribution of air pollutants and subsequently affected communities of airborne bacteria and fungi, distinctly. In addition, our results found that abundant species were more sensitive than rare taxa in response to environmental changes associated with Chinese New Year (CNY). The relative abundance of potential bacterial pathogens in the bacterial community was higher during CNY compared with those before and after CNY, which indicated that human activities during CNY increase the threats of airborne microbes to human beings.

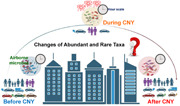

## INTRODUCTION

Microbes in the near‐surface atmosphere ranging from 10^4^ to 10^6^ cells/m^3^ pose potential health effects on human well‐being [1]. Files and his colleagues have summarized the differences in the abundance and diversity of airborne microbes between urban and rural and further revealed that rural airborne microbiomes were healthier due to the higher abundance and diverse bacteria and fungi [2]. However, the pathogens in the air would threat human health and cause a series of diseases [3] by colonizing on the skin, mucous membranes, and digestive and respiratory tracts. Potential microbial pathogens are ubiquitous in the air, and numerous studies have reported many pathogenic bacteria and fungi isolated from air and dust [4]. Diverse and complex compositions of airborne microbial communities and their potential risks for human health have been explored due to the pursuit of human beings for better air quality. In theory, airborne microbes should be assessed based on active air sampling due to the large spatial and temporal variations [5]. However, most of the recent studies explored the microbial communities in the air through short‐term sampling or dust collection nowadays, which cannot capture overall microbial situations, and so far, there have been few studies reporting the dynamics of airborne microorganisms at high time resolution, such as hourly time series. Such hour‐scale characterizations of airborne microbial composition community would provide the potential to better understand the relationship between airborne microbes and atmospheric changes.

The microbial community in an environment is normally composed of a few taxa with high abundance (i.e., abundant species) and more taxa with low abundance (i.e., rare species). In recent years, an increasing body of research has found the differences in functional characteristics [6, 7] between abundant and rare taxa, and rare species have recently gained increasing attention due to their ecological importance [8]. Abundant taxa are the most active category driving biogeochemical cycles [9, 10] and present greater survivability to the changing environments due to their high abundance [11], while rare taxa always serve as a reservoir of genetic and functional diversity for the whole community and contribute to the robustness of microbial community. This means that rare taxa would respond promptly to environmental disturbance to maintain community stability. A previous study has demonstrated conditionally rare taxa with 1.5%−28% of total community membership over time accounted for up to 97% of temporal variability in microbial communities in different ecosystems, for example, air, soil, and water [12], and numerous studies have also indicated that some rare species would turn to be dominant in response to changes of environments [13, 14, 15]. Besides, distinct changes have been found for abundant and rare microbial subcommunities in response to different environments. For example, Shade and his colleagues indicated the contribution of conditionally rare taxa to the community dissimilarity of airborne differed among time [12]. Meanwhile, increasing studies have demonstrated differentiation strategies of abundant and rare taxa in response to environmental changes in soil [9, 16] and aquatic [17, 18] ecosystems. However, recent investigations about abundant and rare species mainly focused on soil and aquatic ecosystems, but their patterns in the atmosphere are still unclear. Local meteorological parameters, air quality, and environmental pollutants have been indicated as important factors in affecting the distribution of airborne bacteria [19], and anthropogenic activities would increase the proportion of potential pathogens in urban areas [20]. Previous studies have demonstrated the differences in the abundance and community structure of airborne microorganisms between haze and nonhaze day [21, 22, 23], and Zhen et al. indicated a stronger impact of meteorological factors on airborne bacteria compared with air pollutants [24]. Chinese New Year (CNY), with significant differences in populations and human activities (e.g., traffic and setting off fireworks) from non‐CNY periods, is the most important holiday for Chinese people and usually lasts about 7 days. A previous study has demonstrated the “holiday effect” of CNY on air pollutants. That is, the concentrations of NO_x_, CO, SO_2_, and PM10 in the air of Taipei metropolitan area were significantly lower in CNY than those in non‐CNY, but the variation in the concentration of O_3_ was reversed [25]. However, the knowledge about the responses of airborne microbes, especially abundant and rare taxa, to atmospheric changes related to CNY is still limited.

The microbial community assembly, focusing on the contribution of stochastic (e.g., birth, death, and immigration) and deterministic (e.g., environmental factors and species interactions) processes to shaping the compositions and abundance of taxa in ecological communities, is a key issue in evaluating the influence of environmental changes on the microbial communities, which may in turn affect their functioning. Recent studies quantifying the importance of stochastic and deterministic processes for microbial community assembly mainly focused on soil [16, 26] and water [27, 28] environments, but limited studies reported the assembly of microbial community in the air or dust. Li et al. [29] have demonstrated a higher contribution of stochastic processes in shaping bacterial and fungal community assembly in urban dust. Nevertheless, the community assembly of abundant and rare taxa might be predominated by different processes due to their distinct ecological responses to environmental changes [30] and would impact their function in different ecosystems. However, so far, there is no study to explore the response of the community assembly of airborne abundant and rare taxa to environmental changes associated with CNY.

In this study, we collected the airborne bacteria and fungi at a minimum of 2‐h resolution to characterize the airborne microbial communities with the aims to: (i) clarify the high time‐resolved dynamics of abundant and rare microbial (both bacterial and fungal) taxa; (ii) reveal the effects of environmental changes associated with CNY on the composition community and microbial assembly of the abundant and rare subcommunities in the air; and (iii) assess the potential threats of airborne microbes to human health at different periods based on potential bacterial pathogens in the atmosphere.

## RESULTS

### Temporal dynamics of airborne bacteria and fungi

**Figure 1:**
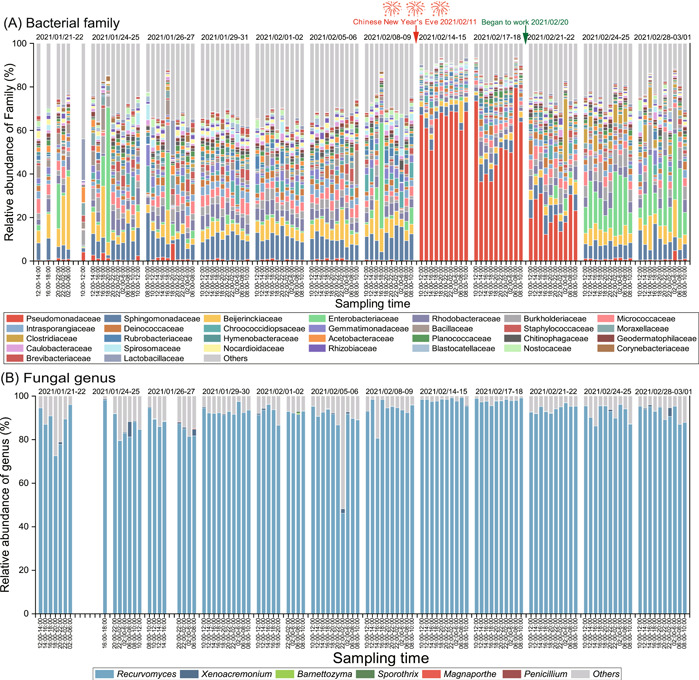
Hourly scale dynamics of bacteria and fungi. (A) Community compositions of bacterial family. (B) Community compositions of fungal genus. In this study, the airborne microbes were collected from January 01, 2021 to March 01, 2021. In this year, the Chinese New Year (CNY) began from February 02, 2021 to February 17, 2021. At the sampling site, staffs began to work on February 20, 2021.

**Figure 2:**
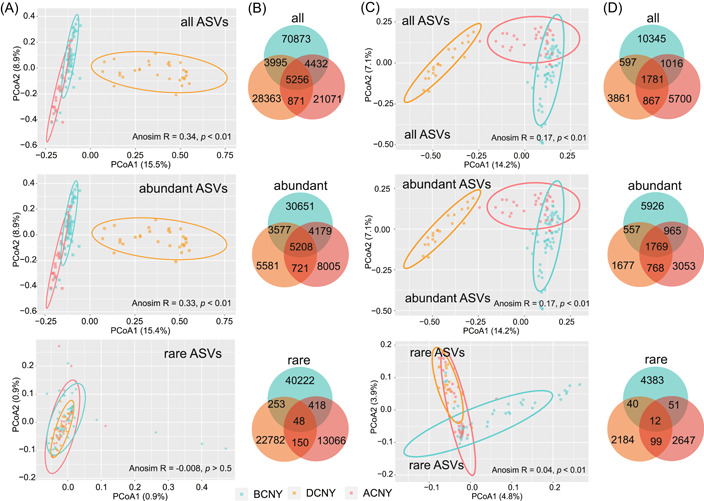
Changes in community patterns of microbes in response to the atmospheric changes associated with Chinese New Year (CNY). (A) Principal coordinates analysis presenting the patterns of airborne bacteria based on Bray–Curtis distances. (B) Venn diagram showing the numbers of unique and shared bacterial amplicon sequence variants (ASVs) among the three periods. (C) Principal coordinates analysis presenting the patterns of airborne fungi based on Bray–Curtis distances. (D) Venn diagram showing the numbers of unique and shared fungal ASVs among the three periods. The difference in microbial communities among the three periods was detected by the anosim test. ACNY, after Chinese New Year; BCNY, before Chinese New Year; DCNY, during Chinese New Year.

A total of 10,932,267 bacterial sequences (55,558–116,133 for each sample) and 5,502,530 fungal sequences (24,409–74,450 for each sample) were obtained in this study. The collected samples were clustered strongly by time and into three groups: before CNY (BCNY), during CNY (DCNY), and after CNY (ACNY) (Figures 1 and 2A,B). Proteobacteria was always the phylum with the highest relative abundance in bacterial communities, followed by Actinobacteria, Firmicutes, and Bacteroidetes (Supporting Information: Figure S1A). Dothideomycetes was the dominant class (46.2%–99.3%) in fungal communities (Supporting Information: Figure S1B). The families of Pseudomonadaceae, Sphingomonadaceae, Beijerinckiaceae, and Enterobacteriaceae predominated in the bacterial communities (Figure 1A), and the genera of *Recurvomyces* affiliated within Dothideomycetes was dominant in the fugal communities (Figure 1B). The family of Pseudomonadeceae significantly (*p* < 0.01) increased during CNY but markedly decreased after CNY. Enterobacteriaceae significantly (*p* < 0.01) increased after CNY (Figure 1A). The significant (*p* < 0.01) effects of CNY on fungal communities were also observed in this study (Figures 1B and 2C).

All and abundant taxa showed significantly differed community structures among different time groups, that is, BCNY, DCNY, and ACNY (Figure 2A,C, anosim *p* < 0.01), while the rare bacterial taxa had a similar community structure (*p* > 0.05, Figure 2A). The distances of bacterial communities between BCNY and DCNY and between DCNY and ACNY were more far than that between BCNY and ACNY based on all and abundant taxa (*p* < 0.001, Supporting Information: Figure S2A,B), while the distances between DCNY and ACNY were more less than that between BCNY and ACNY for fungal communities (*p* < 0.001, Supporting Information: Figure S2C,D). Venn plots indicated that most of the bacterial and fungal amplicon sequence variants (ASVs) were classified as the group‐specific ASVs, and most (99.1% for bacteria and 99.3% for fungi) of the shared ASVs among the three groups belonged to abundant taxa (Figure 2B,D).

Our results showed a significantly (*p* < 0.01) lower α‐diversity for abundant bacteria of DCNY, while a significantly (*p* < 0.01) higher Shannon index for rare bacterial taxa of DCNY compared to BCNY (Supporting Information: Figure S3). For fungal communities, the Shannon indexes for all (Supporting Information: Figure S4B) and abundant (Supporting Information: Figure S4D) fungal communities of DCNY were significantly (*p* < 0.001) lower than those of BCNY and ACNY, while the Shannon index for rare fungi (Supporting Information: Figure S4F) of BCNY was significantly lower (*p* < 0.05) than those of DCNY and ACNY. There was no significant difference in the Chao1 index for fungal communities (Supporting Information: Figure S4A,C,E) among the three groups.

**Figure 3:**
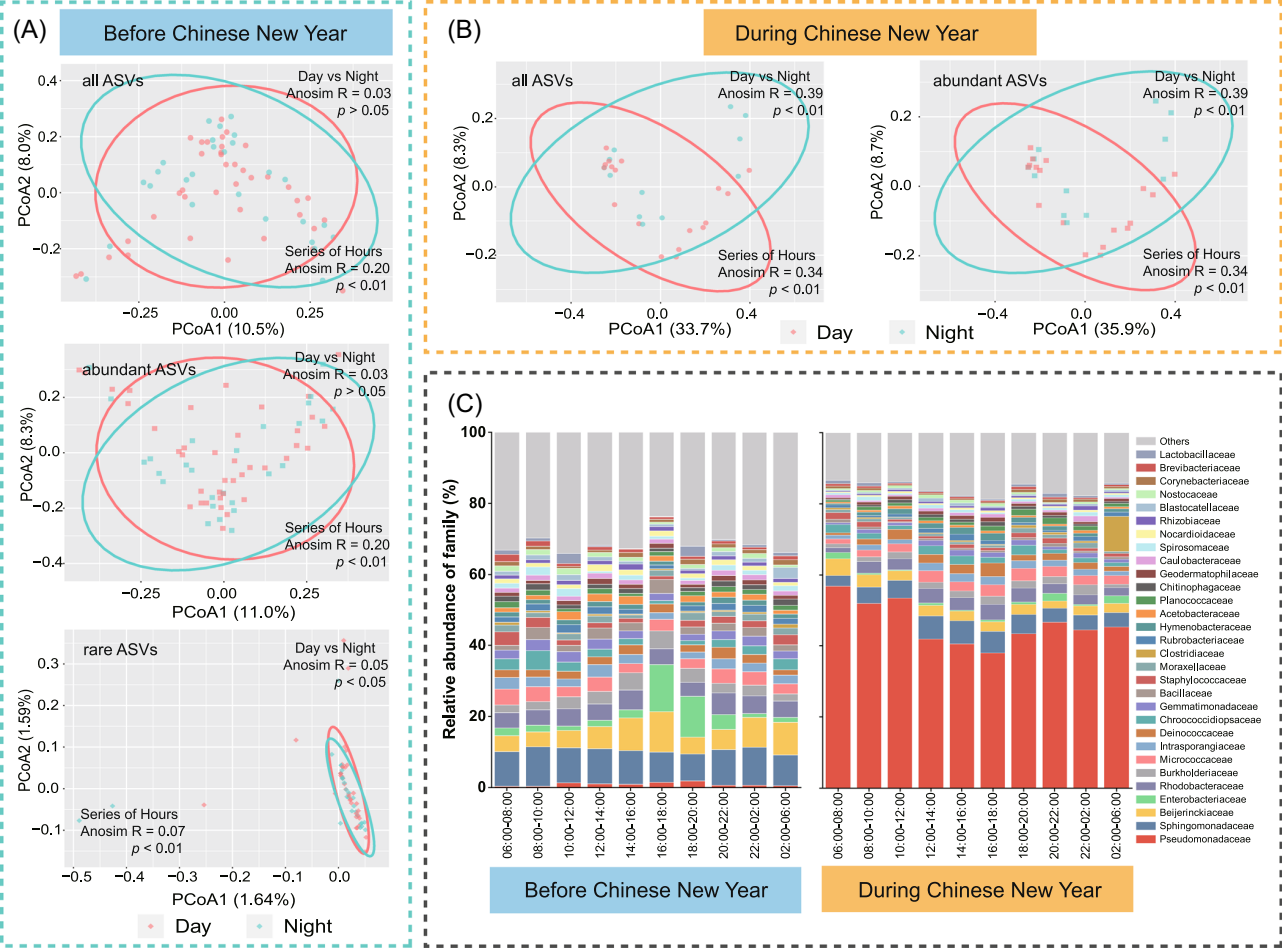
Distribution patterns of airborne bacterial communities before and during Chinese New Year (CNY). (A) Principal coordinates analysis (PCoA) patterns of airborne bacterial all, abundant, and rare subcommunities before CNY. Day/night did not significantly affect the patterns of all and abundant subcommunities, but rare subcommunities. Hour time series significantly (*p* < 0.01) affected all, abundant, and rare subcommunities. (B) PCoA patterns of airborne bacterial all and abundant subcommunities during CNY. Day/night and hour time series significantly (*p* < 0.01) affected the communities of all and abundant taxa. (C) Hour‐scale dynamics of bacterial compositions (family level) before and during CNY.

We further found a significant (*p* < 0.01) difference in abundant (Figure 3B) but not rare (Supporting Information: Figure S5B) bacterial subcommunity between night and day during CNY, while the effects of day/night on both abundant and rare subcommunities were reversed before CNY (Figure 3A). Hour‐scale changes in abundant bacterial subcommunities were observed before and during CNY (Figure 3A, B), but the hour time series only significantly (*p* < 0.01) impacted the communities of rare taxa before CNY (Figure 3A). The variation in bacterial communities among hours might be attributed to the changes in Pseudomonadaceae and Enterobacteriaceae before CNY and to the changes in Pseudomonadaceae during CNY. Before CNY, the relative abundance of Pseudomonadaceae was higher at 10:00–20:00 compared to other times, and the Enterobacteriaceae increased significantly (*p* < 0.01) at 16:00–20:00 (Figure 3C). The changes of the two families differed from their changes during CNY, where Pseudomonadaceae decreased from 12:00 to 18:00 and Enterobacteriaceae increased at 20:00–6:00 (Figure 3C). After CNY, both day/night and hour time series did not significantly affect the communities of airborne bacteria (Supporting Information: Figure S5). Apart from bacterial communities, only abundant fungal subcommunities before CNY were affected by day/night and hour time series (Supporting Information: Figure S6).

### Taxa with significant difference among time groups

**Figure 4:**
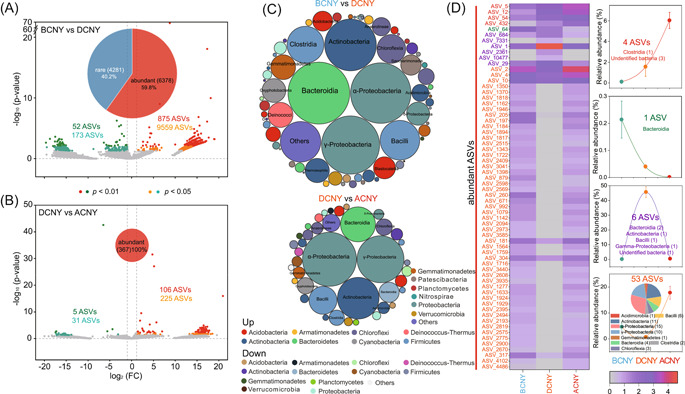
Changed bacterial amplicon sequence variants (ASVs) with significant difference in the atmosphere between BCNY and DCNY and between DCNY and ACNY. (A) ASVs with significant difference between BCNY and DCNY; (B) ASVs with significant difference between DCNY and ACNY. (C) Classification of the ASVs with significant differences between BCNY and DCNY and between DCNY and ACNY. (D) Bacterial ASVs with regular changes during different periods (i.e., significantly increased or decreased from BCNY to ACNY, significantly higher or lower during CNY compared with BCNY and ACNY). Warm colors in plots (A) and (B) indicated the bacterial ASVs with significant decrease during CNY compared with BCNY (A) and after CNY compared with DCNY (B), respectively, while cold colors indicated the bacterial ASVs with significant increase during CNY compared with BCNY (A) and after CNY compared with DCNY (B), respectively. The bubble size in plot (C) indicated numbers of the changed ASVs affiliated within this taxon. The showed relative abundance in heatmap (D), showing the changed ASVs with regular changes during different periods, was converted by log_e_ (average relative abundance of ASVs in bacterial community times 10000+1). The line plot following heatmap (D) showing the changes of changed bacterial ASVs among different time groups. The relative abundance of the total changed bacterial ASVs with regular changes differed significant (*p* < 0.05) among time groups. ACNY, after Chinese New Year; BCNY, before Chinese New Year; DCNY, during Chinese New Year.

Compared to BCNY, a total of 10,659 ASVs significantly changed, with 225 ASVs increase and 10,434 decrease in airborne bacterial communities of DCNY. In which, 59.8% was grouped as the abundant taxa (Figure 4A). Whereas only 367 ASVs significantly changed between DCNY and ACNY, and all of them were grouped as the abundant taxa (Figure 4B). Most of the changed ASVs were affiliated within the classes of α‐Proteobacteria, γ‐Proteobacteria, Actinobacteria, Bacteroidia, and Bacilli (Figure 4C). Four abundant bacterial ASVs (one ASVs affiliated within Clostridia and three ASVs affiliated within unidentified bacteria) increased from BCNY to ACNY, while one abundant ASVs affiliated within Bacteroidia was reserved (Figure 4D). Compared with BCNY and ACNY, six abundant ASVs were significantly enriched but 53 abundant ASVs significantly decreased during CNY (Figure 4D). For fungal communities, most of the changed ASVs either between BCNY and DCNY (Supporting Information: Figure S7A) or between DCNY and ACNY (Supporting Information: Figure S7B) were identified as the abundant taxa and affiliated within the genera of *Recurvomyces* and unidentified fungi (Supporting Information: Figure S7C).

### Temporal changes in environmental factors

The environmental factors, including meteorological parameters, metals, non‐metals, oxides, ions, and volatile organic compounds (VOCs), are listed in Supporting Information: Table S1. The principal component analysis (PCA) results showed a strong difference in the patterns of environmental factors among time groups (anosim test *p* < 0.001). The samples of BCNY separated from other samples at PCA1, while DCNY samples differentiated from other samples at PCA3 (Supporting Information: Figure S8). We further found that Zn, Mn, Hg, NO_3_^−^, NH_4_^+^, PM2.5, PM10, VOCs, and AVOCs (anthropogenic VOCs) were significantly (*p* < 0.05) higher in BCNY than those in other groups (Supporting Information: Figure S9), while the concentrations of Cu, Ba, K, O_3_, and K^+^ were higher in DCNY samples (Supporting Information: Figure S9). The non‐AVOC (non‐anthropogenic VOCs), Sb, Na^+^, CO, and NO_2_ were significantly enriched in the atmospheric samples of ACNY (Supporting Information: Figure S9).

### Factors related to the variation of the airborne microbial communities

**Figure 5:**
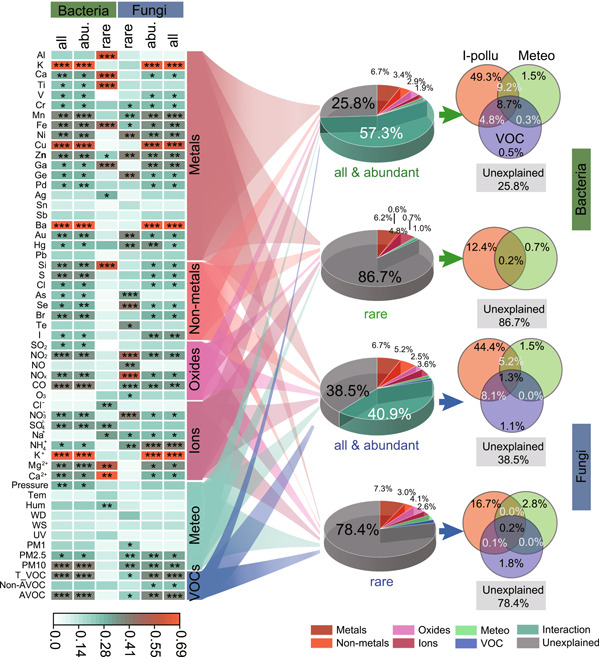
Contribution of environmental factors to variation of microbial communities. I‐Pollu: Inorganic pollutants, including metals, non‐metals, oxides, and ions; Meteo: meteorological parameters. *, **, and *** indicating significant impact with *p* < 0.05, *p* < 0.01, and *p* < 0.001, respectively.

Most of the detected environmental factors affected the structures of bacterial and fungal communities, especially the subcommunities of abundant taxa (Figure 5). The pRDA further showed that environmental factors, respectively, explained 74.2% and 61.5% variations of abundant bacterial and fungal subcommunities while contributed only 13.3% and 21.6% variations of rare bacterial and fungal subcommunities, respectively. Among the environmental factors, inorganic pollutants played the most important role in shaping the communities of both bacteria and fungi, followed by meteorological parameters and VOCs.

**Figure 6:**
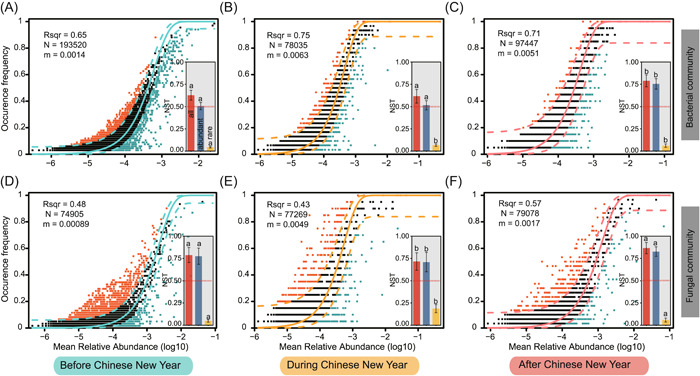
Community assembly of airborne abundant bacteria and fungi. (A–C) neutral community model (NCM) showing the community assembly of abundant bacteria collected before Chinese New Year (CNY), during CNY, and after CNY, respectively. (D–F) Community assembly of abundant fungi collected at different times. Bar plots showing the normalized stochastic ratio (NST) represented the relative contribution of stochastic processes to the community assembly of all, abundant, and rare microbes. Lowercase letters indicated a significant difference among the three different periods. The NST value > 0.50 indicates that stochastic processes predominated in regulating the community assembly of microbes.

A large fraction of relationship between the occurrence frequency of abundant ASVs (i.e., bacterial abundant ASVs and fungal abundant ASVs) and their relative abundance variations (Figure 6) was estimated by the neutral community model (NCM), but the community assembly of rare ASVs (i.e., bacterial rare ASVs and fungal rare ASVs) did not fit to NCM (*R* ^2^ < 0) (Supporting Information: Figure S10). Further, the NCM showed a goodness of fit for abundant bacteria with 65% (Figure 6A), 75% (Figure 6B), and 71% (Figure 6C) of explained community variance for BCNY, DCNY, and ACNY, respectively. The goodness of fit to neutral model was better for bacteria than for fungi (Figure 6D–F). Most of the normalized stochastic ratio (NST) value was higher than 50% boundary point for the subcommunities of both abundant bacteria and fungi, while it was significantly lower than 50% boundary point for the rare subcommunities (Figure 6 bar plots). These results indicated a more important role of stochastic processes in contributing to the assembly of abundant microbial subcommunities, but deterministic processes contributed more to the assembly of rare microbial subcommunities.

### Distribution and relative abundance of potential pathogenic bacteria to human

**Figure 7:**
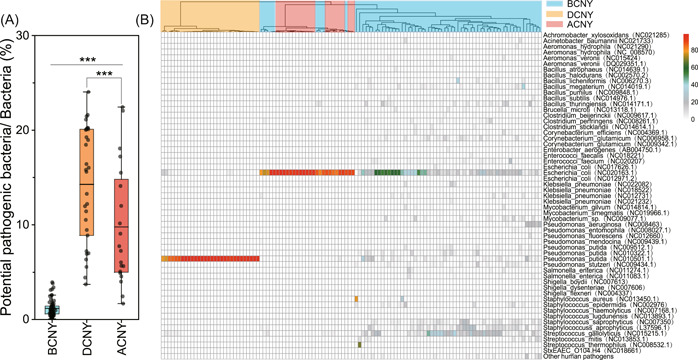
Relative abundance of potential bacterial pathogens in the atmosphere. (A) The relative abundance of potential bacterial pathogens in bacterial communities; (B) heatmap showing the relative abundance of each pathogen in bacterial pathogenic communities. *** Indicating significant difference with *p* < 0.001.

Potential pathogenic bacteria were detected in all samples and a total of 120 potential pathogens (Supporting Information: Table S2) were identified across all samples. The relative abundance of potential pathogens in bacterial communities ranged from 0.07% to 24.85%, with a highest average abundance in samples of DCNY (14.28%) followed by ACNY (9.78%) and BCNY (1.17%) (Figure 7A). The heatmap showed that the community patterns of potential pathogens were clustered by time groups (Figure 7B). Compared with BCNY, *Pseudomonas putida* enriched in the samples of DCNY, while *Escherichia coli* were higher in the samples of ACNY.

## DISCUSSION

The phylum of Proteobacteria, Actinobacteria, Firmicutes, and Bacteroidetes predominated in the bacterial communities from the air samples of this study, which was supported by previous research [5, 20, 31, 32]. We found a significant increase of Pseudomonadeceae in the air during CNY compared to those before and after CNY and a lowest abundance of Pseudomonadeceae at 12:00–18:00 during CNY. This phenomenon might be partially explained by the sudden increase in air temperature due to selection of firework on Pseudomonadeceae during CNY. A previous study has demonstrated the positive effect of heat on the relative abundance of Pseudomonadeceae in the air [33]. In addition to fireworks, the atmospheric changes associated with commuting might be another possible reason affecting the relative abundance of Pseudomonadeceae, which was supported by the increase of Pseudomonadeceae at 10:00–12:00 and 16:00–20:00 before CNY and previous studies indicating the dominance of *Pseudomonas* and *Acinetobacter* in bacterial communities in outdoor environments with heavy traffic [34, 35]. The family of Enterobacteriaceae was lower during CNY than before CNY, which might be partially explained by the lower people density during CNY. Xiamen is a city with a large amount of external population, which leads to fewer human activities during CNY in comparison with BCNY due to returning home. We further found that Enterobacteriaceae increased at 16:00–20:00 before CNY and at 20:00–6:00 during CNY. One possible reason was the increase of domestic sewage and garbage, which have been indicated as important factors influencing the relative abundance of Enterobacteriaceae in the atmosphere [36, 37, 38]. The class of Dothideomycetes was dominant in the airborne fungal communities, which was consistent with the results of an earlier study [39] reporting a dominant position of Dothideomycetes in dry depositions.

Recent studies have illustrated the great importance of rare microbial subcommunity to ecological functions in different ecosystems [8, 9, 11]. Thus, the rare and abundant microbial communities were comparatively analyzed in this study. Abundant bacterial and fungal subcommunities were significantly split by the time, but there was no significant difference in rare bacterial subcommunities among the three periods. In addition, the Bray–Curtis distances between DCNY and other periods were more far than that between BCNY and ACNY, which were further supported by the Venn plot showing more shared abundant ASVs between BCNY and ACNY. Further, we found only abundant bacterial subcommunity were significantly (*p* < 0.01) affected by day/night and hour time series during CNY. No significant difference in rare fungal subcommunity was observed before CNY. Significant (*p* < 0.01) effects of hour time series on abundant bacterial and fungal subcommunities before CNY were observed. These results indicated the distinct responses of the abundant and rare microbial subcommunities in the atmosphere to the environmental changes related to the CNY, which are consistent with several recent studies reporting differentiation strategies of rare and abundant microbial taxa in response to changing environmental factors in different ecosystems, including air [12], soil [9, 40], and water [41, 42]. For instance, a previous study about airborne microbes indicated that conditionally rare taxa would be abundant, and their dynamics explained a large fraction of the community dissimilarity when they were abundant [12]. Moreover, in this study, we found that the majority of changed ASVs were grouped into abundant subcommunities, suggesting that rare subcommunities were more stable in response to the atmospheric changes associated with CNY.

Generally, abundant members are thought as most active and important in the biogeochemical cycles [43] but rare members act as a seed bank that may become active under favorable conditions [12, 44] for maintaining microbial stability and functioning in various ecosystems. Abundant members are suggested to be more sensitive to changes in environmental factors in comparison with rare taxa. In our study, the variations of abundant microbial (i.e., bacteria and fungi) subcommunities were largely explained by environmental factors, especially inorganic pollutants, while the detected environmental factors only explained 13.3%–21.6% variations of rare microbial subcommunities. These results are consistent with several recent studies indicating that rare subcommunities were more stable compared to abundant subcommunities under environmental pressure [8, 9, 40] and suggesting the different ways rare and abundant communities respond to environmental changes. In our study, inorganic pollutants played a more important role than meteorological parameters and VOCs in the distributions of both abundant and rare taxa. This finding suggested the critical position of human activities in affecting the communities of airborne microbes [20] due to fireworks and other human activities that have been indicated as important drivers in changing air quality [45, 46], especially water‐soluble ions and elements [47].

In addition to the difference between abundant and rare microbial taxa, previous studies [48, 49, 50] have revealed the distinct responses of bacteria and fungi to environmental changes. In this study, the variations of all bacterial communities (*R* = 0.34) among the three groups were greater than those of all fungal communities (*R* = 0.17) based on the anosim test, supported by the Venn plots. For example, only 9.1% of all bacterial ASVs were shared between BCNY and ACNY, but 13.7% of all fungal ASVs were shared between them. These results verified that bacteria were more sensitive than fungi to the surrounding changes although rare bacterial subcommunities were not significantly affected by CNY. A study has indicated a more sensitive bacterial rate in the air of healthcare settings compared to fungi [51]. In soil ecosystems, bacterial communities also have been demonstrated to be more sensitive than fungal communities to changes in soil properties [50, 52]. This might be partially explained by the difference in cell walls between bacteria and fungi. Some bacteria, particularly gram‐positive bacteria with peptidoglycan cell walls, can resist environmental changes and survive in various ecosystems. Further, fungal chitinous cell walls would enhance their resistance to changes in surrounding environments [53]. Another possible reason is the existence of fungal spores, which have been demonstrated as important states to adapt to environmental changes [54, 55, 56] and maintain fungal community and diversity.

Apart from environmental factors, the community assembly also plays critical roles in regulating the community, diversity, and functioning of microbes. Our results revealed that the stochastic process predominated in the community assembly of both abundant bacteria and abundant fungi, indicating the distribution of abundant bacteria and abundant fungi in the air was mainly shaped by passive dispersal, microbial birth and death [57], and ecology drift [58]. However, we found higher contributions of the deterministic process to the community assembly of both rare bacteria and rare fungi, suggesting that inherent abiotic (e.g., heavy metals, pollutants, and pH of particles) and biotic factors (e.g., interactions between microbes) in the atmosphere shape the subcommunities of rare microbes. Our study further found that the contributions of the stochastic process to the community assembly of both abundant and rare microbes significantly differed among BCNY, CNY, and ACNY, suggesting activities associated with CNY and work would change the microbial community assembly, supported by a previous study [29] indicating spatiotemporal variations in dust‐associated bacterial and fungal community assembly due to changes in environmental factors and microbial interactions. In this study, despite a successful prediction of the distribution of most of the abundant microbial ASVs, a considerable portion of abundant ASVs were still unpredictable by neutral processes. This result indicated that deterministic processes such as microbial interactions and environmental factors could not be neglected in regulating the community assembly of bacteria and fungi in the atmosphere.

Potential bacterial pathogens were detected across all samples, indicating the potential threats of airborne bacteria to human health. The percentage of potential pathogens in bacterial communities ranged from 0.07% to 24.85%, with an average of 6.22%, which was comparable with that detected in urban air collected from street area [32]. We found that the relative abundance of bacterial pathogens increased with CNY and then decreased after CNY. Meanwhile, the patterns of potential pathogens differed among the three periods. These results suggested that human activities during CNY significantly affected the potential health risk of airborne microbial pollution by changing the distribution and abundance of potential bacterial pathogens in the air, supported by numerous studies indicating the significant impact of human activity on patterns and abundance of microbes [20], especially pathogens [59, 60] in the atmosphere. Staggeringly, the relative abundance of bacterial pathogens reached highly to 24.85%, with an average of 14.28% in the air during CNY, which was comparable with that detected on the skin of human beings [61] and even higher than those detected in the air of hospitals [5]. These results suggested that airborne microbes may threaten human health due to the presence of pathogens, and human activities associated with CNY would increase this threat.

## CONCLUSION

In summary, the effects of atmospheric changes related to CNY on abundant and rare subcommunities were determined, and the changes in pattern and relative abundance of potential bacterial pathogens in response to environmental changes associated with CNY were observed in this study. Distinct responses of abundant and rare subcommunities to atmospheric changes associated with CNY were detected, and abundant taxa were more sensitive to the environmental changes associated with CNY. In addition, we found that stochastic processes contributed more to the community assembly of abundant microbes, while deterministic processes predominated in regulating the community assembly of rare taxa. Potential bacterial pathogens were detected in all air samples, and the ratio of potential pathogens to bacteria was significantly higher during CNY, indicating human activities associated with CNY would increase the potential threats of microbes in the air to human health. These findings prompt the necessity of controlling human activities during CNY and provide some scientific basis for the improvement of air quality.

## METHODS

### Sampling site and collection of airborne microbes

Airborne microbes were sampled from 20 January to 1 March 2021 at the roof of the main building of the Institute of Urban Environment (118°4′12.50″E, 24°36′52.47″N), Chinese Academy Sciences (IUE, CAS) in Xiamen, where a regional atmospheric supersite has been established for surveillance of hourly scale environmental factors including meteorological parameters and pollutants (e.g., tropospheric ozone, VOCs, oxides, and metals). The main building of IUE is near a street and a bay, and the sampling point is about 150 m from the street and the bay, respectively. The meteorological parameters and the concentrations of air pollutants were determined according to previous studies [62, 63]. Three replicates were collected for each sampling time.

Particulate matters (PMs) from a total of 120 m^3^ air (flow rate 1 m^3^/min) were collected in a 90 mm diameter cultural plate with 2 mL of mineral oil (Sigma) using a High Vol Portable Bioaerosol Sampler (P‐1000, Beijing Dingblue Technology Co., Ltd) at about 1.5 m height above the roof. The time series of sampling included 10:00–12:00, 12:00–14:00, 14:00–16:00, 16:00–18:00, 18:00–20:00, 20:00–22:00, 22:00–02:00 (collected for 2 h), 02:00–06:00 (collected for 2 h), 06:00–08:00, and 08:00–10:00 and are detailed in Supporting Information: Table S1 and Figure 1. The collected PMs were eluted into a 15‐mL tube using 2 mL 0.01 M phosphate buffer solution (PBS) with 2% Tween20 (Beyotime, China) and centrifuged at 14,000 × *g* for 5 min. The pellets were used for DNA extraction.

### DNA extraction, target‐gene amplification, and sequencing

Microbial DNA was extracted using a QIAamp® BiOstic® Bacteremia DNA kit (QIAGEN) according to the provided protocol with some modifications. Briefly, 200 μL of MBL and 250 μL self‐provided enzymatic lysis buffer (20 nM Tris, 2 mM Na_2_‐EDTA, 1.2% TritonX‐100, 20 mg/mL lysozyme, and pH 8.0) were added to resuspend the pellets and lyse the collected microbial cells. DNA extractions were then conducted in accordance with the manufacturer's instructions. Finally, a total of 50 μL of solution elution buffer was used to elute the purified DNA. The extracted DNA was stored at −20°C before target‐gene amplification.

To explore the community compositions of airborne bacteria and fungi, bacterial 16S rRNA genes and fungal ITS2 regions were amplified using 338F/806R [64] and ITS3/ITS4 [65], respectively. A total of 50 μL PCR amplification mixtures contained 25 μL of Premix Taq™ Mix (TaKaRa), 0.2 μM of forward primer, 0.2 μM of reverse primer, 3.0 μL of DNA template, and nuclease‐free water. The PCR was processed as previously described [5], and amplicons were gel‐purified using a universal DNA purification kit (DP214, Tiangen, Beijing). Purified amplicons were sent to Magigene Biotechnology Co. for sequencing using an Illumina Nova6000 platform.

### Data processing and accession numbers

The obtained sequences were checked using DADA2 [66], and low‐quality sequences were discarded. The resulting high‐quality sequences were analyzed using Quantitative Insights Into Microbial Ecology version 2 (QIIME2) [67]. For both bacteria and fungi, ASVs were generated with a 97% similarity, and ASVs with only one sequence were discarded in the following analysis. Silva (v138) and Unite (v8.2) were used for the classification of the bacterial and fungal taxa, respectively. ASVs identified as mitochondria and chloroplast sequences were removed. The checked ASVs were used to calculate α‐diversity of microbial communities using QIIME2. To identify the potential human bacterial pathogens, the high‐quality sequences were blasted against reference sequences reported in a previous study [68] with an identity threshold >99% and an *E*‐value < 10^−10^.

### Definition of abundant and rare taxa

To explore the dynamics of abundant (AT) and rare taxa (RT) community structures and their responses to environmental factors associated with CNY, a local relative abundance threshold of 1% for abundant taxa and 0.01% for rare taxa [69] was set in this study. We grouped bacterial and fungal communities into six categories of taxa, including (i) always abundant taxa (AAT), (ii) conditionally abundant taxa (CAT), (iii) conditionally abundant or rare taxa (CRAT), (iv) moderate taxa (MT), (v) conditionally rare taxa (CRT), and (vi) rare taxa (RT) as previous studies reported [17, 70]. The ASVs with relative abundance >1% in at least one sample (i.e., AAT, CAT, and CRAT) were combined as abundant taxa in this study [70].

### Statistical analysis

The principal coordinates analysis (PCoA) patterns of microbial communities were determined based on Bray–Curtis distance using R software with the “vegan” package. The contribution of each air pollutant and meteorological parameter to the variances in distribution of environmental factors among different groups were determined by PCA using R software with the package of “factoextra.” The anosim test was used to test the differences in patterns of microbes and environmental factors among different groups. The partial redundancy analysis (pRDA) was used to analyze the relative contribution of each environmental factor to the variances of microbial communities in the air. The ASVs with fold change >2 between different groups were identified as changed taxa associated with CNY using DESeq. 2 software. The heatmap for potential bacteria was visualized in according with a previous study [71]. NCMs were constructed to determine the assembly mechanisms for the microbial communities in air samples using R software [72]. In addition, the normalized stochasticity ratio (NST) was calculated to determine the contribution of the stochastic process to the community assembly of microbes using R software with the package of “NST” [73]. If necessary, one‐way analysis of variance and the correlation test were performed using SPSS 20.0 (SPSS, Inc), and figures were plotted using OriginLab 2021 in this study.

## AUTHOR CONTRIBUTIONS

Hu Li, Jian‐Qiang Su, and Yong‐Guan Zhu designed this study. Hu Li, You‐Wei Hong, Meng‐Ke Gao, and Xin‐Li An performed the experiments. Hu Li and Xiao‐Ru Yang performed bioinformatics and statistical analyses. Hu Li and You‐Wei Hong interpreted the data and wrote the manuscript. All of the other authors revised and edited the manuscript. All authors read and approved the final manuscript.

## CONFLICT OF INTEREST STATEMENT

The authors declare no conflict of interest.

## Supporting information

Supporting information.

Supporting information.

## Data Availability

The sequences collected in this study have been deposited in the Science Data Bank using the following links for bacteria (10.57760/sciencedb.09044) and fungi (10.57760/sciencedb.09057). The script for the definition of abundant and rare taxa used in this study is available at https://github.com/IUEhli/iMeta2023-1. Supplementary materials (figures, tables, graphical abstract, slides, and videos), Chinese translated version and update materials may be found in the online DOI or iMeta Science http://www.imeta.science/.
