# Supplementary material for: Distinct responses of airborne abundant and rare microbial communities to atmospheric changes associated with Chinese New Year
Source: Imeta. 2023 Oct 11;2(4):e140. doi: 10.1002/imt2.140 (PMC10989829; doi:10.1002/imt2.140)
Supplement: Supplementary file 1 — Supporting information. [file IMT2-2-e140-s002.docx]

**Distinct responses of airborne abundant and rare microbial communities to atmospheric changes associated with Chinese New Year**

Hu Li ^1, 5 #^ *, You-Wei Hong ^1, 2, 5 #^, Meng-Ke Gao ^1, 3^, Xin-Li An ^1, 5^, Xiao-Ru Yang ^1, 5^, Yong-Guan Zhu ^1, 4, 5^, Jin-Sheng Chen ^1, 2, 5^, Jian-Qiang Su ^1, 5^ *

^1^ Fujian Key Laboratory of Watershed Ecology, Key Laboratory of Urban Environment and Health, Institute of Urban Environment, Chinese Academy of Sciences, Xiamen, 361021, China

^2^ CAS Center for Excellence in Regional Atmospheric Environment, Institute of Urban Environment, Chinese Academy of Sciences, Xiamen, 361021, China

^3^ College of Resource and Environmental Science, Fujian Agriculture and Forestry University, Fuzhou 350002, China

^4^ State Key Lab of Urban and Regional Ecology, Research Center for Eco-environmental Sciences, Chinese Academy of Sciences, Beijing 100085, China

^5^ University of Chinese Academy of Sciences, 19A Yuquan Road, Beijing 100049, China

# These authors contributed equally to this study.

Corresponding Author: hli@iue.ac.cn (Hu Li) and [jqsu@iue.ac.cn](mailto:jqsu@iue.ac.cn) (Jian-Qiang Su)

**Running Title:** Patterns and community assembly of airborne microbes during Chinese New Year


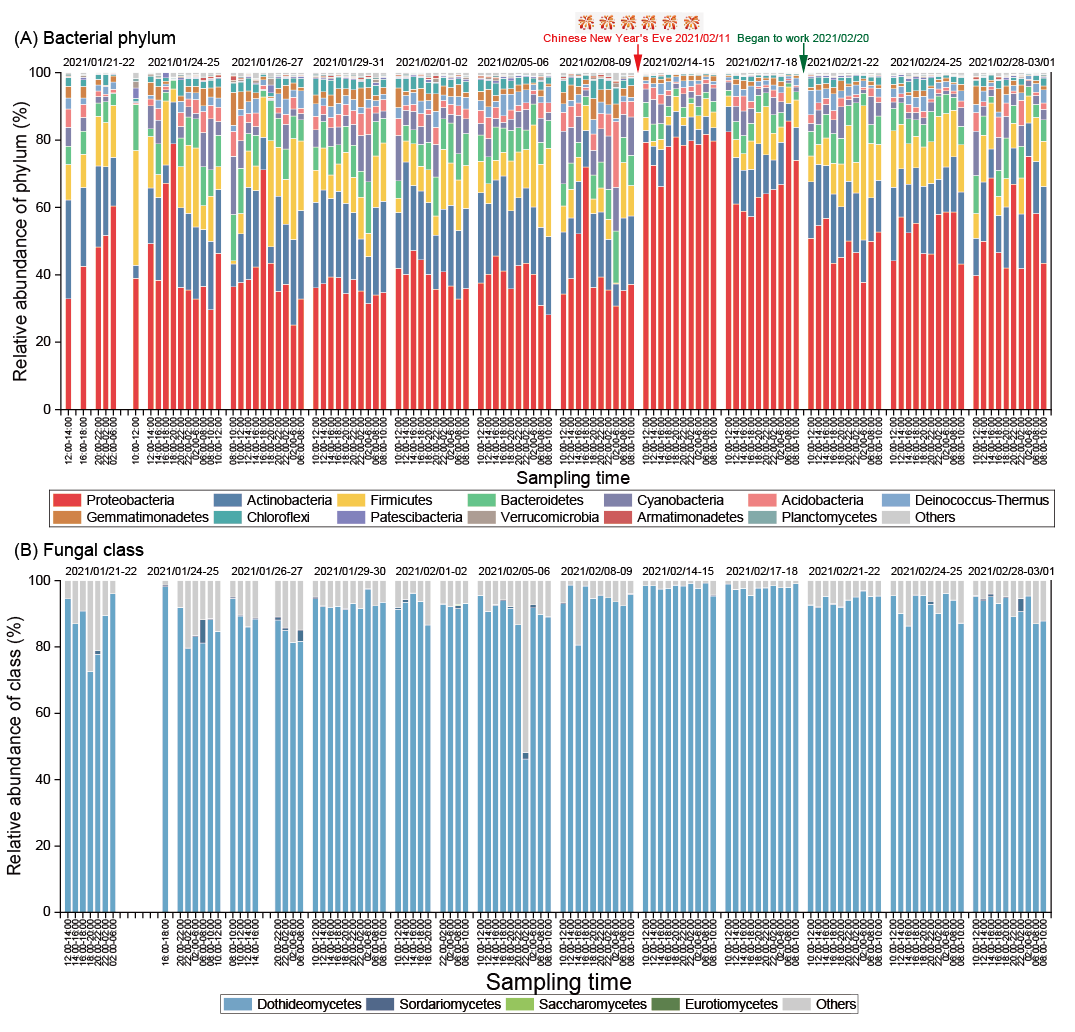


Figure S1 Hourly scale dynamics of bacteria and fungi. (A) community compositions of bacterial phylum. (B) community compositions of fungal class. In this study, the airborne microbes were collected from 2021/01/21 to 2021/03/01. In this year, the Chinese New Year (CNY) began from 2021/02/12 to 2021/02/17. At the sampling site, staffs began to work at 2021/02/20.


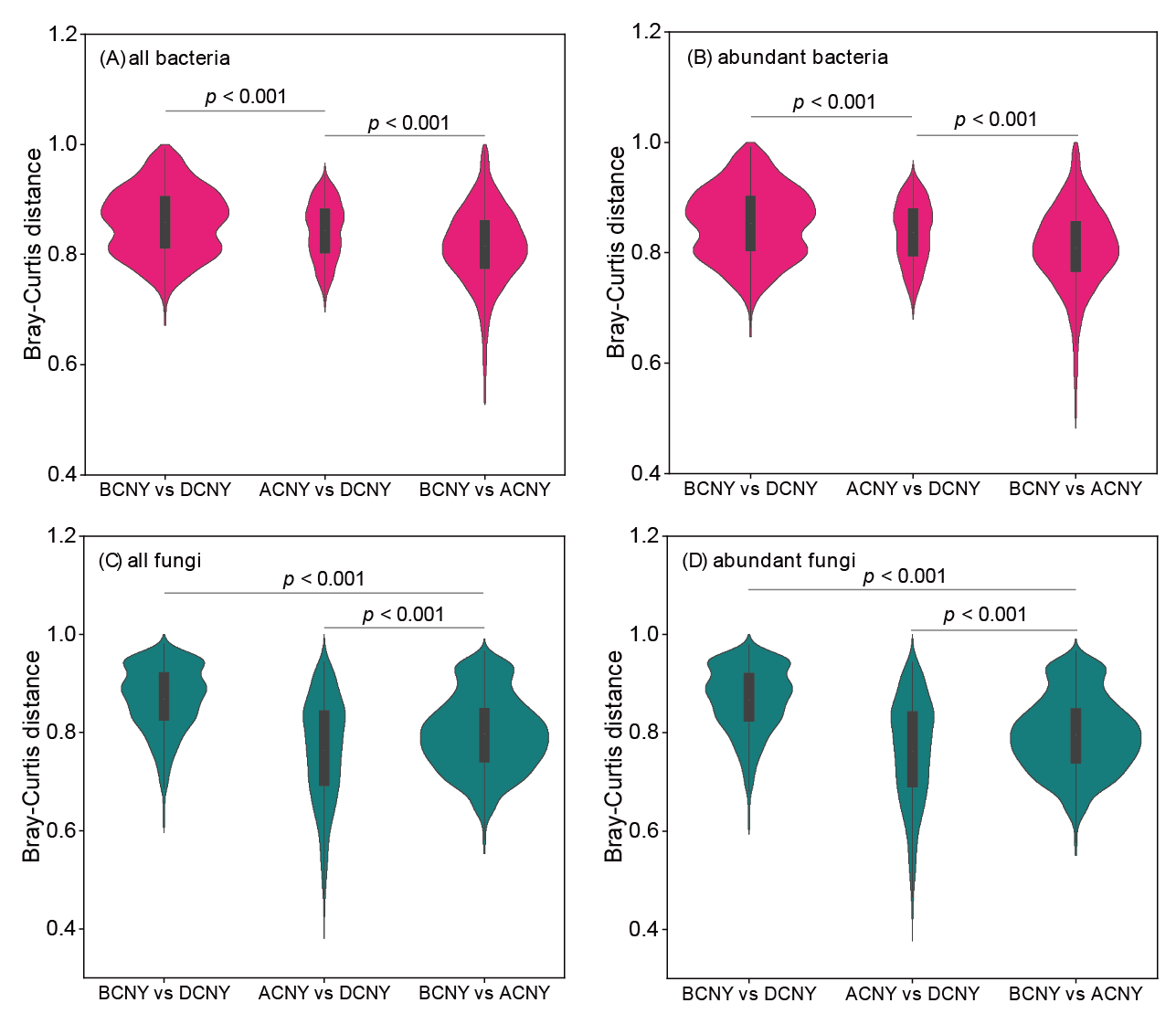


Figure S2 Bray-Curtis distances of microbial communities between different periods. (A, B) Bray-Curtis distances of bacterial communities between different periods based on all and abundant taxa; (C, D) Bray-Curtis distances of fungal communities between different periods based on all and abundant taxa.


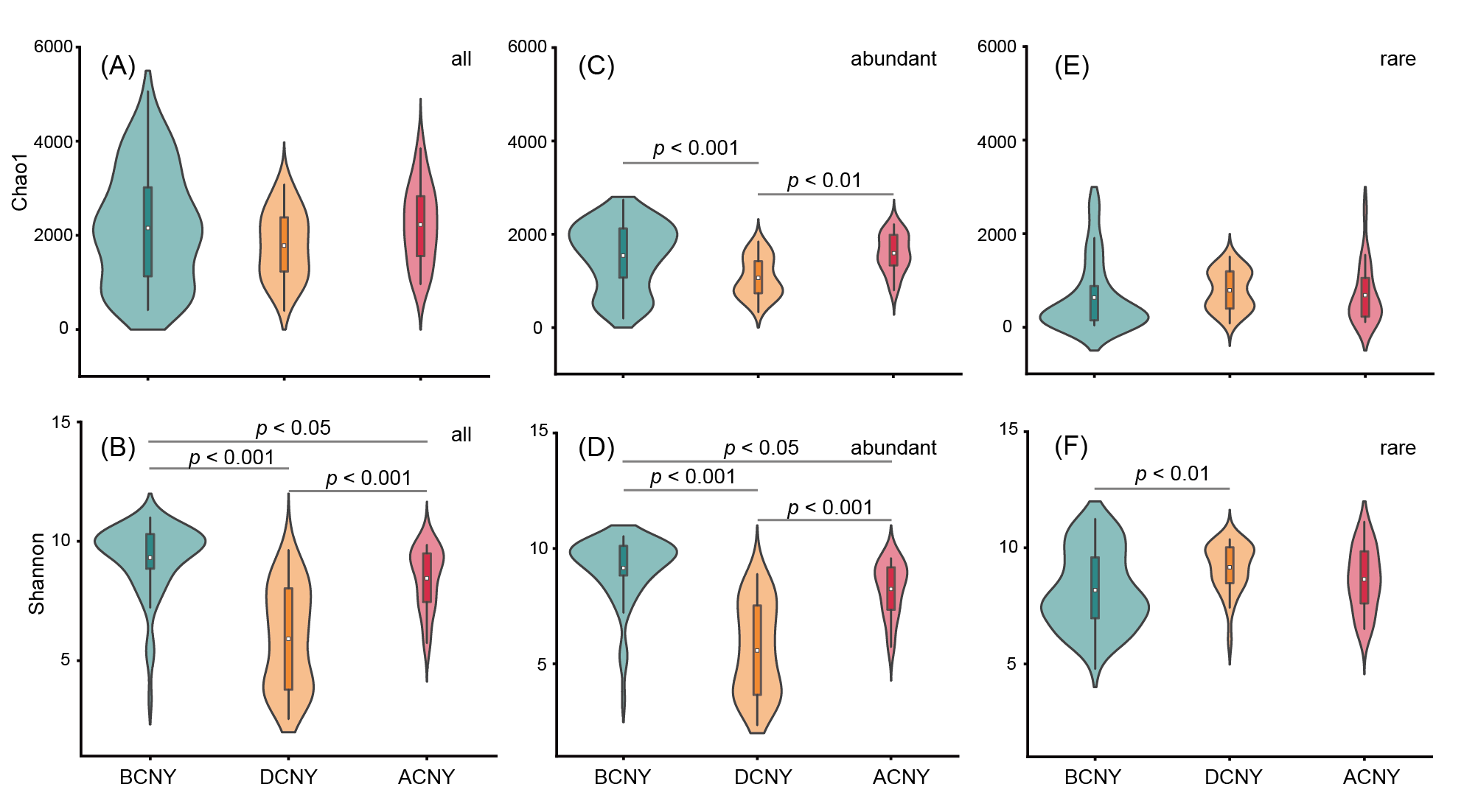


Figure S3 α-diversity of airborne bacterial communities at different periods. (A, C, E) Chao 1 index for all, abundant, and rare bacterial subcommunities, respectively. (B, D, F) Shannon index for all, abundant, and rare bacterial subcommunities, respectively.


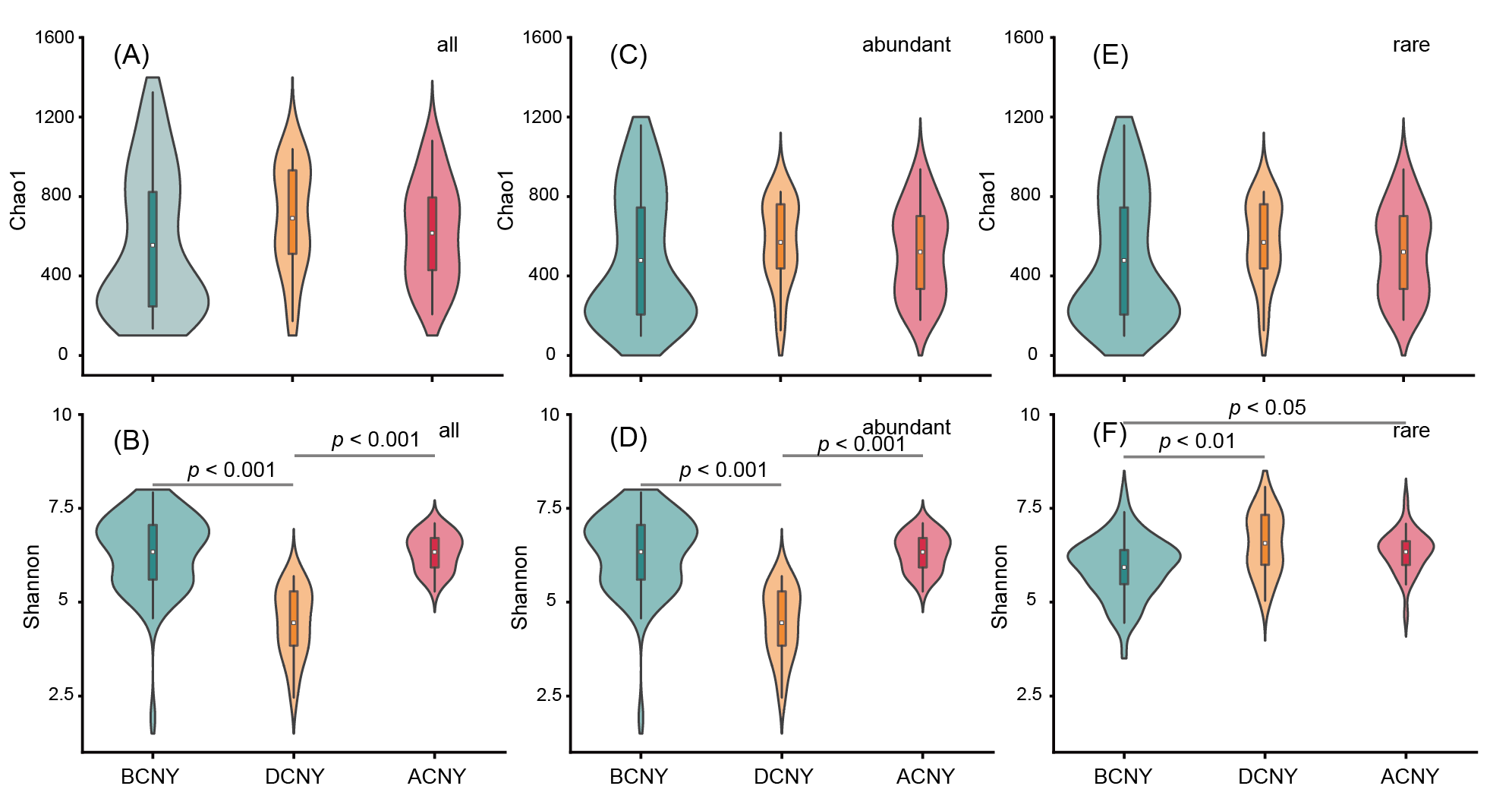


Figure S4 α-diversity of airborne fungal communities at different periods. (A, C, E) Chao 1 index for all, abundant, and rare fungal subcommunities, respectively. (B, D, F) Shannon index for all, abundant, and rare fungal subcommunities, respectively.


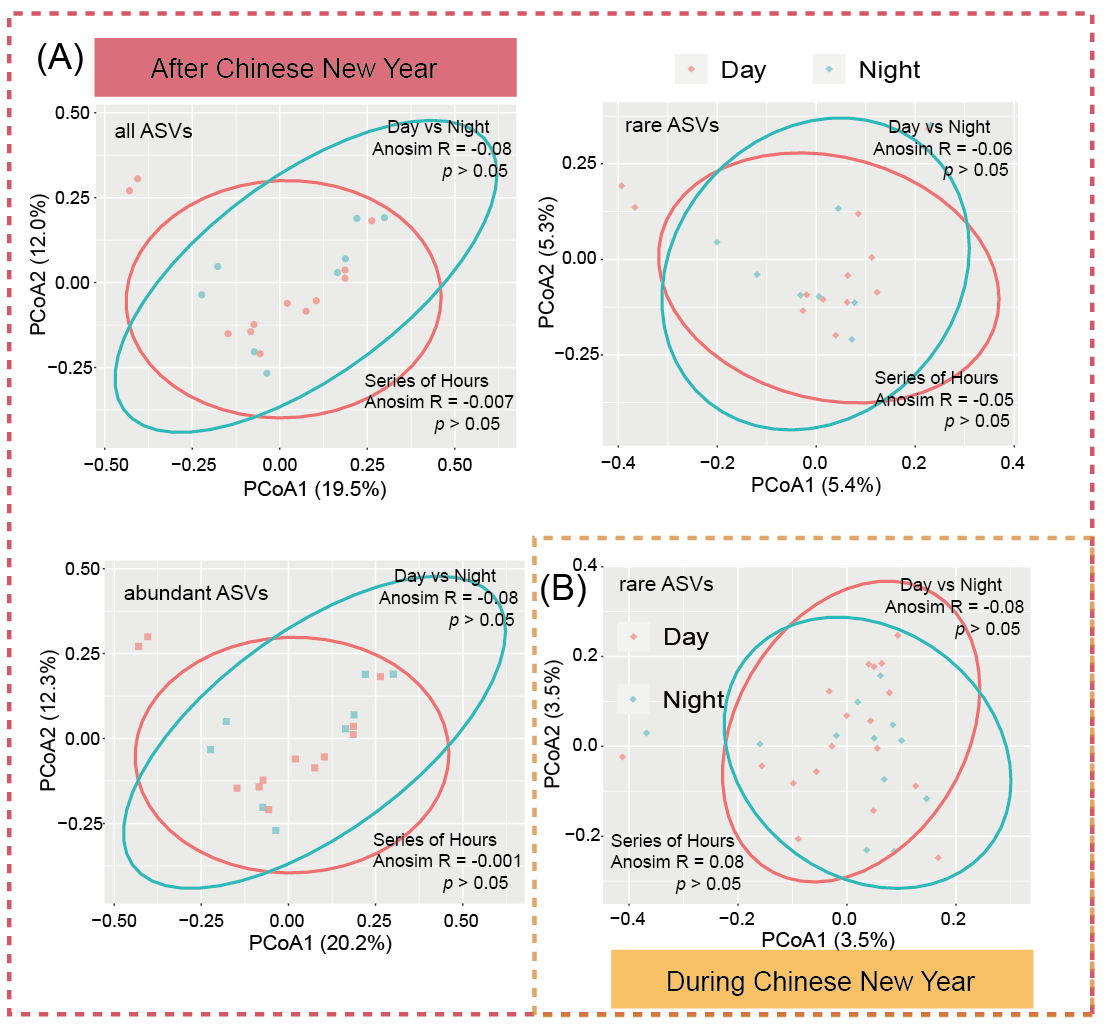


Figure S5 Distribution patterns of airborne bacterial communities after (A) and during (B) CNY. Day/night and hour time series did not significantly (*p* > 0.05) affect the patterns of all, abundant, and rare bacterial subcommunities after CNY (A), and rare subcommunities during CNY (B).


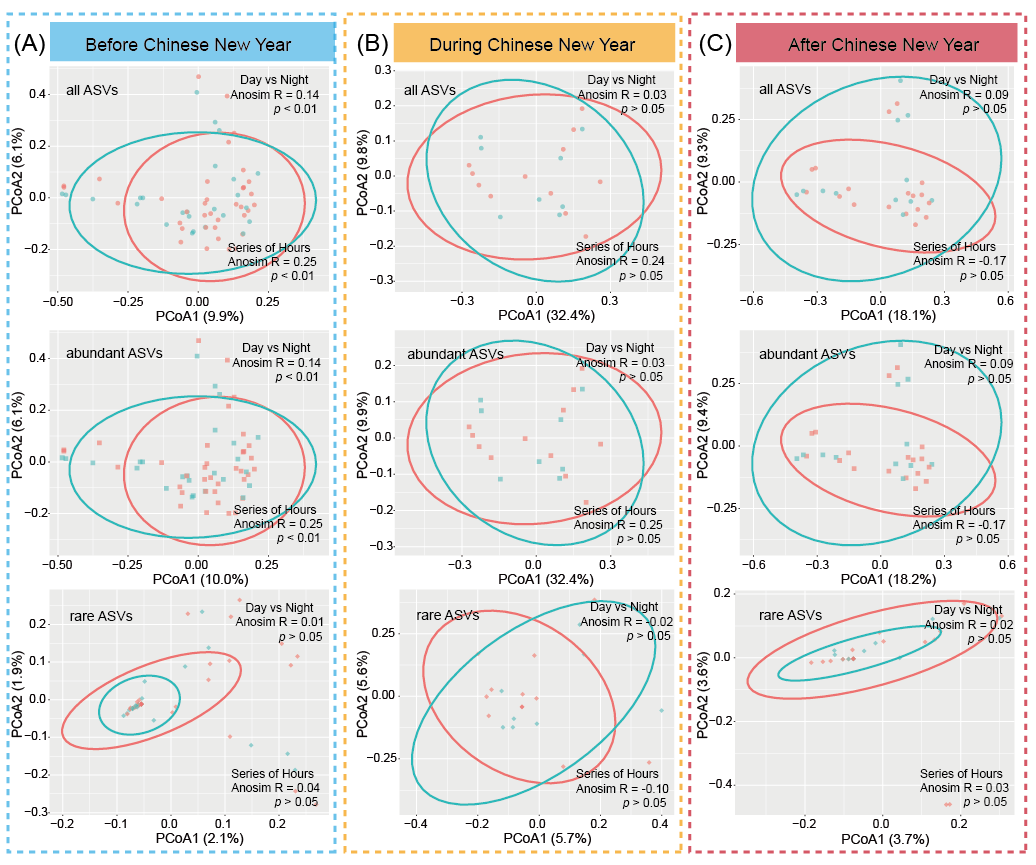


Figure S6 Distribution patterns of airborne fungal communities before (A), during (B), and after (C) CNY. Day/night and hour time series only significantly (*p* < 0.01) affected the patterns of all and abundant subcommunities before CNY.


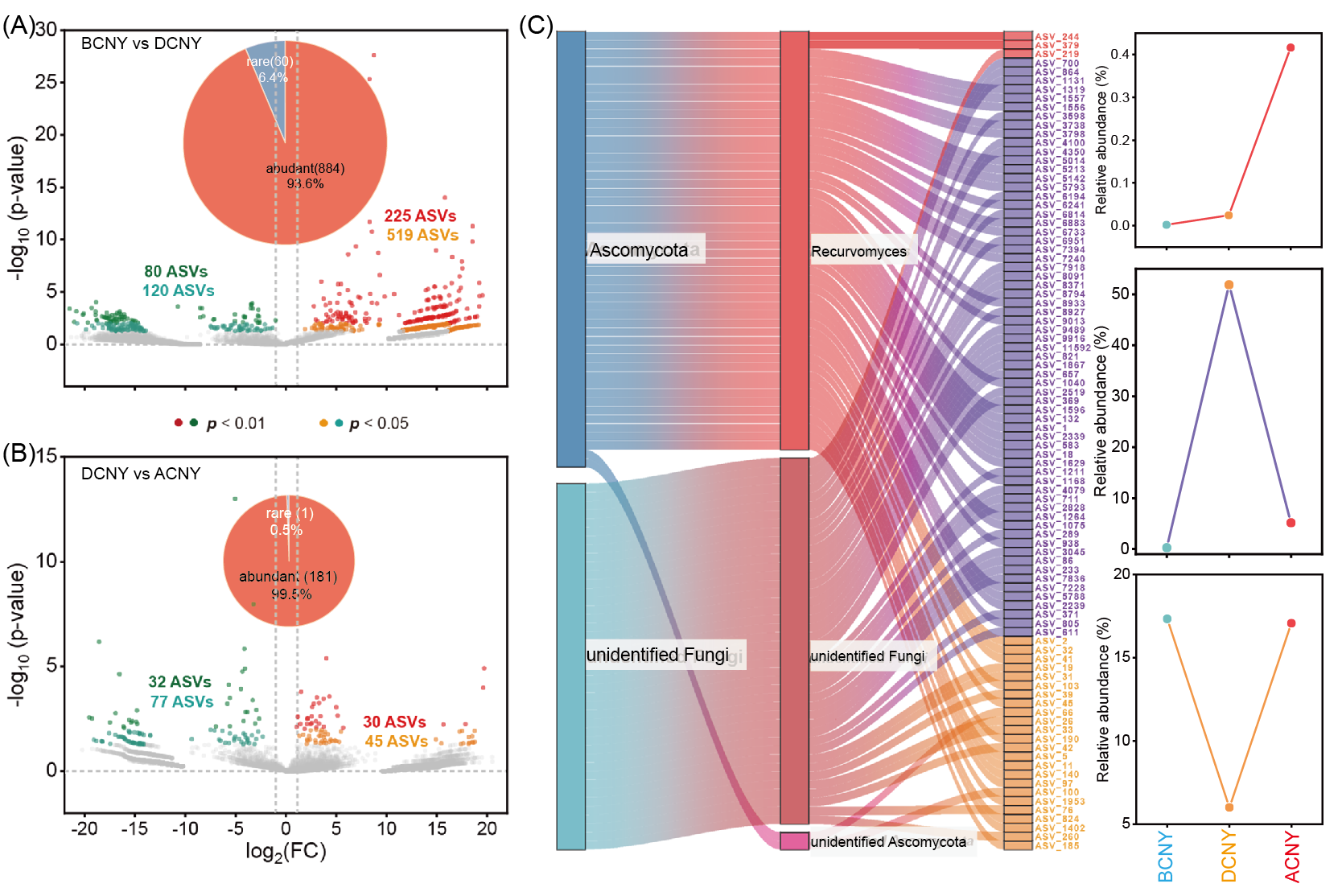


Figure S7 Changed fungal ASVs in the atmosphere between BCNY and DCNY, and between DCNY and ACNY. (A) ASVs with significant difference between BCNY and DCNY; (B) ASVs with significant difference between DCNY and ACNY. (C) Classification of the ASVs with significant differences between BCNY and DCNY, and between DCNY and ACNY. Warm colors in plot (A) and (B) indicated significant increase, while cold colors indicated significant decrease.


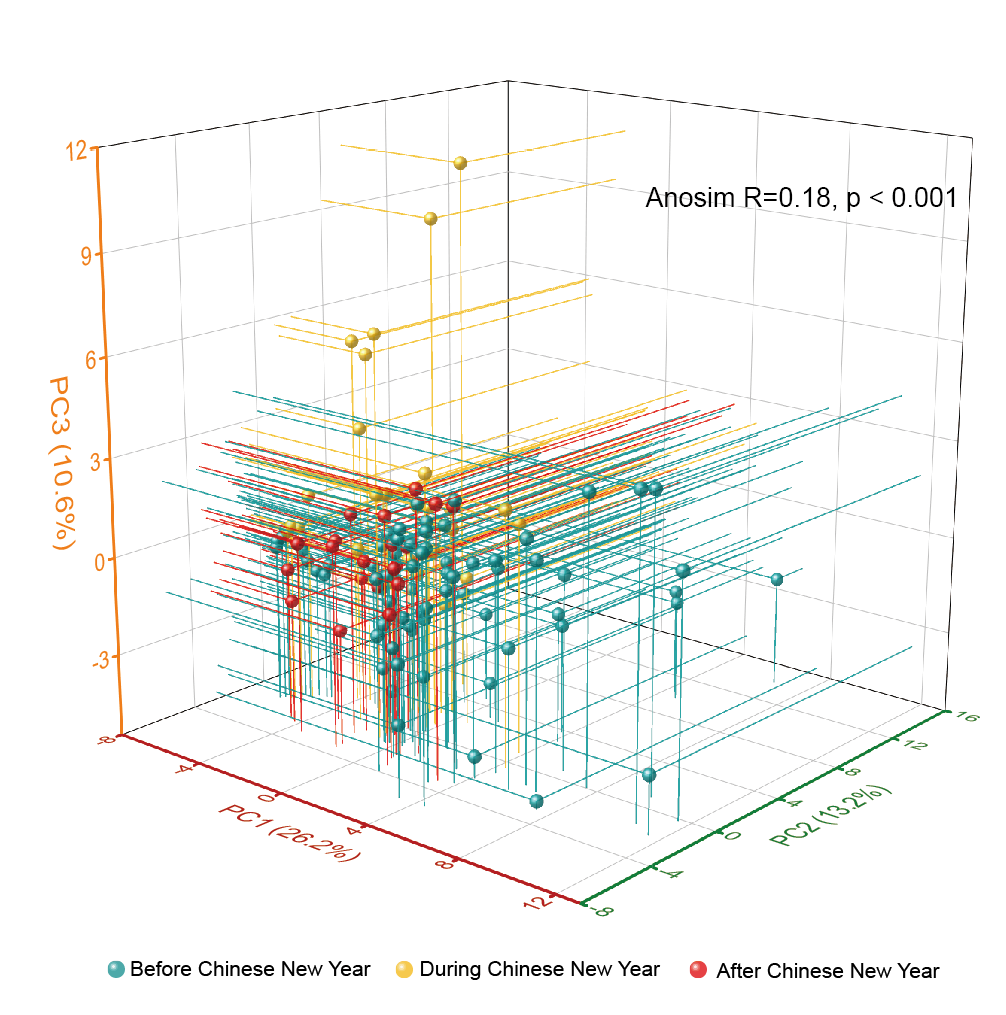


Figure S8 Distribution patterns of airborne environmental factors. PCA (principal component analysis) patterns of air pollutants and meteorological parameters. The atmospheric environment was significantly (*p* < 0.001) different among the time groups.


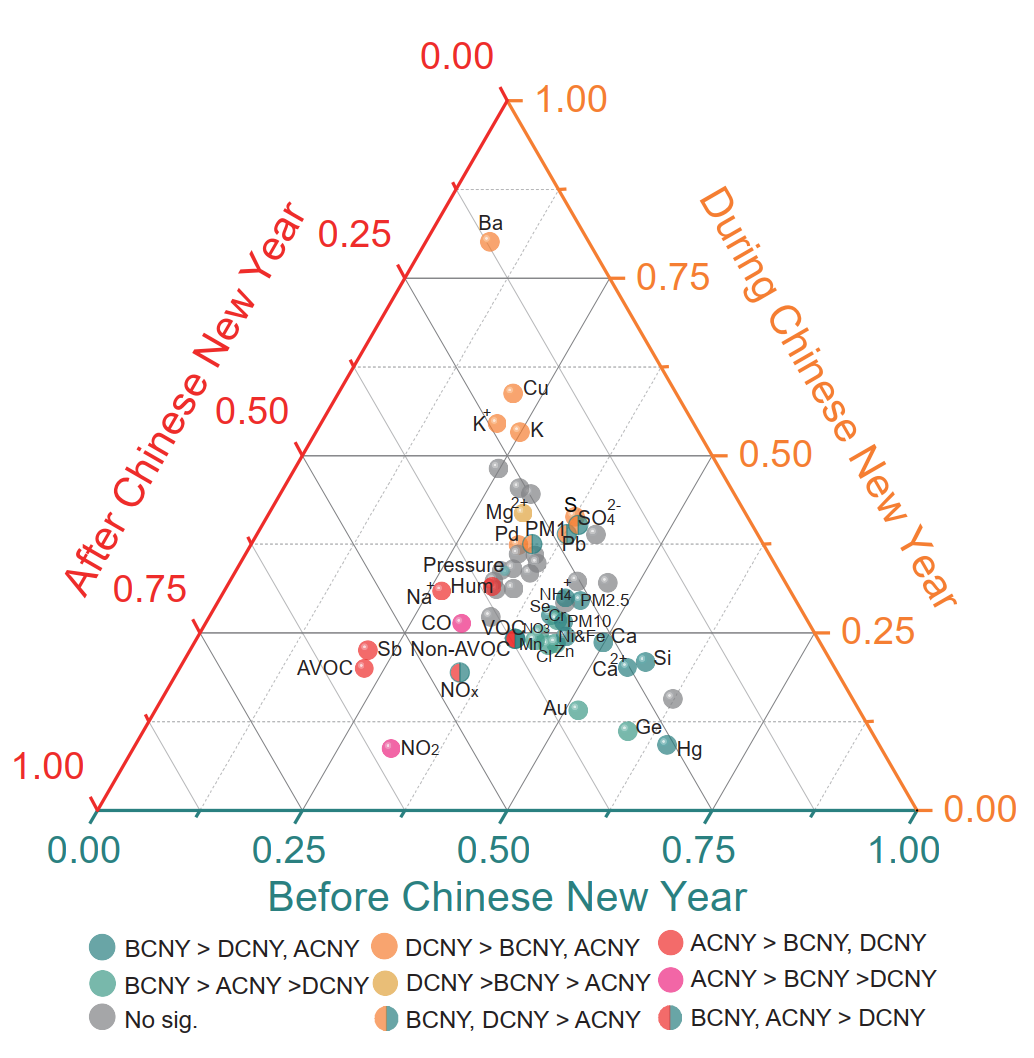


Figure S9 Environmental factors with significant difference among three different periods. BCNY: before Chinese New Year; DCNY: during Chinese New Year; ACNY: after Chinese New Year.


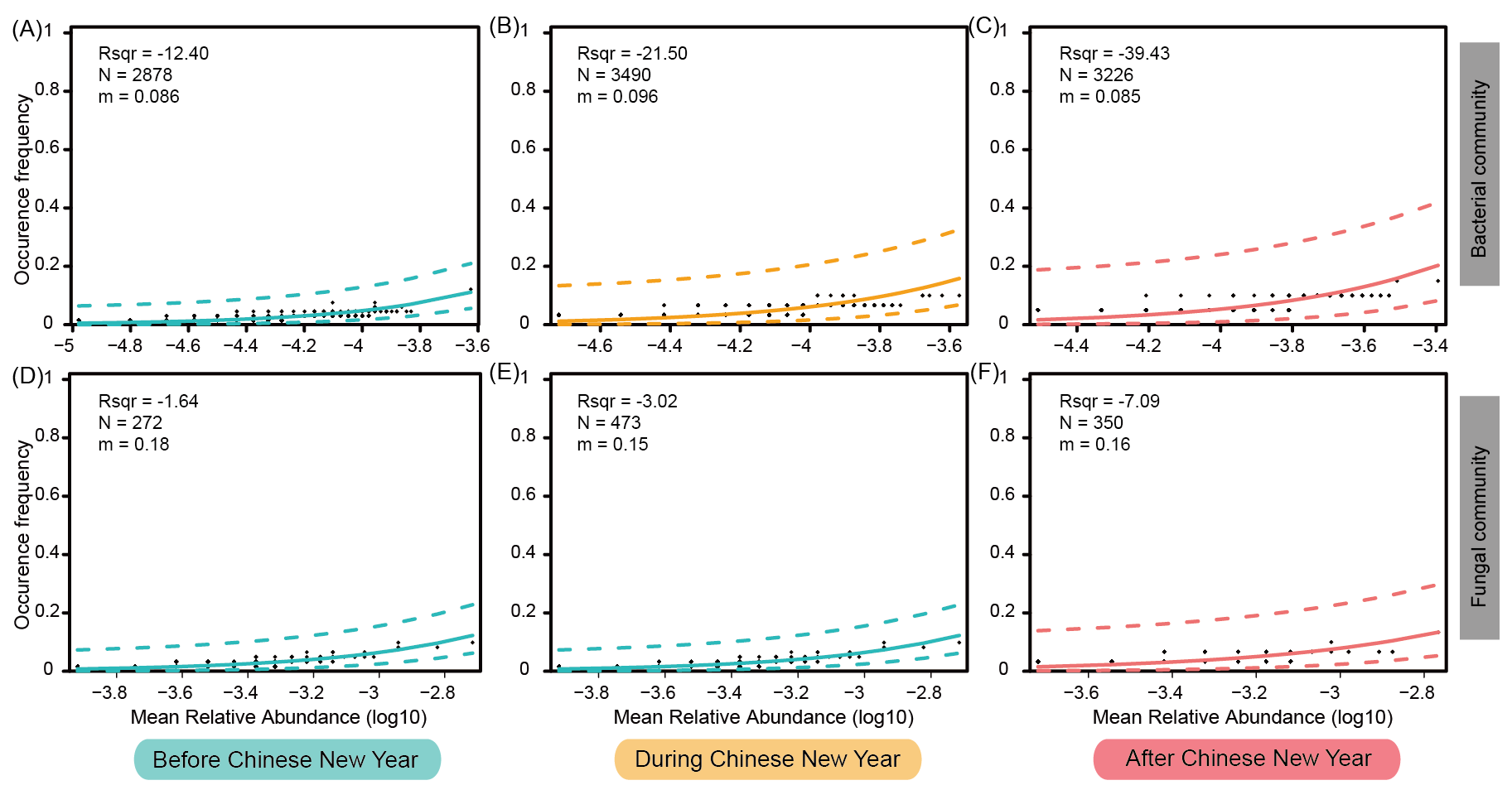


Figure S10 NCM (neutral community model) showing the community assembly of rare bacteria (A-C) and fungi (D-F) in air from different time. The R^2^ < 0 indicated the assembly of rare microbial subcommunities could not predicted well by the NCM.
